# Supplementary material for: Structural and mechanistic basis of proton-coupled metal ion transport in the SLC11/NRAMP family
Source: Nat Commun. 2017 Jan 6;8:14033. doi: 10.1038/ncomms14033 (PMC5230734; doi:10.1038/ncomms14033)
Supplement: Supplementary Information — Supplementary Figures, Supplementary Table. [file ncomms14033-s1.pdf]

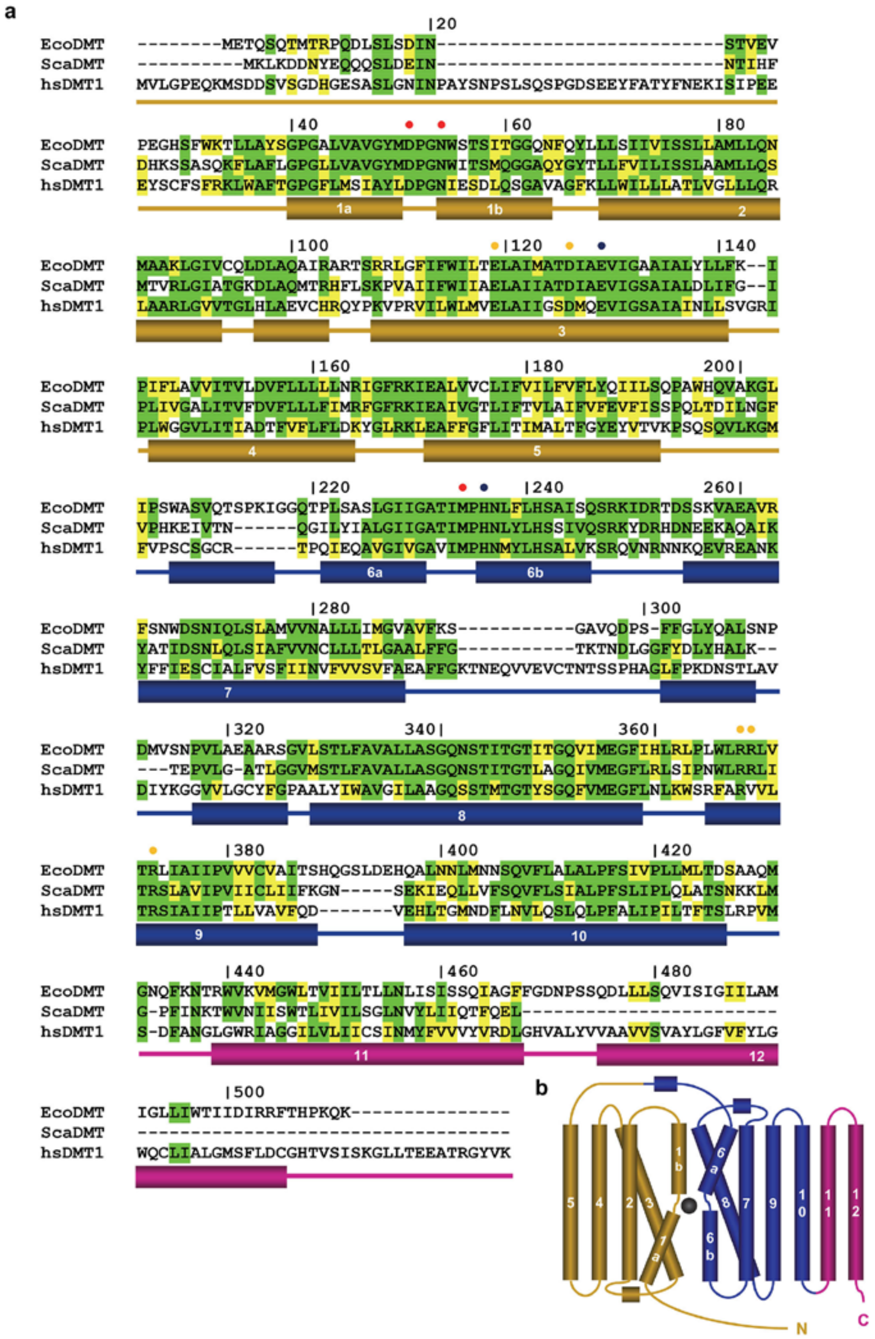

Supplementary Figure 1

EcoDMT sequence and topology.

(**a**) Sequence alignment of EcoDMT (UniProtKB identifier E4KPW4), ScaDMT (UniProtKB identifier A0A178L6Y2-1) and human DMT1 (UniProtKB identifier P49281-2). Identical residues are highlighted in green, similar residues in yellow. Secondary structure elements of EcoDMT are shown below the sequences. The N-terminal domain (α-helices 1–5) is colored in beige, the C-terminal domain (α-helices 6–10) in blue, α-helices 11 and 12 in magenta. Numbers correspond to EcoDMT. Selected residues are labeled: (●) ion-binding site, (●) potential proton acceptors, (●) acidic and basic residues lining a potential intracellular proton exit path. (**b**) Topology of EcoDMT. The color-coding is as in **a**, the cytoplasm is at the bottom, N- and C-termini and numbering of transmembrane helices are indicated.

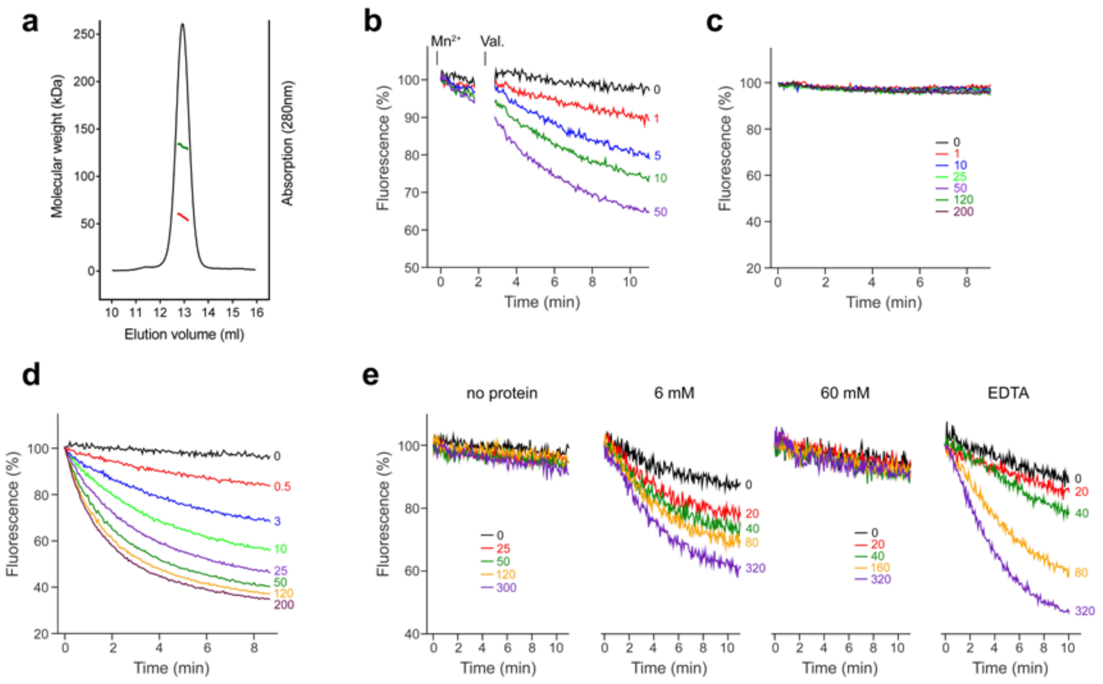

Supplementary Figure 2

Multi-angle light scattering and fluorescence-based transport assays.

(**a**) Multi-angle light scattering coupled to size exclusion chromatography of detergent solubilized EcoDMT. Continuous black traces correspond to the absorption at 280 nm. The molecular weights of the protein-detergent complex (green) and of the protein component (red) obtained from light scattering are shown. The measured molecular weight of the protein (56.9 kDa) is in good agreement with the theoretical molecular weight of monomeric EcoDMT (56.5 kDa). (**b**) EcoDMT mediated Mn^{2+} transport into proteoliposomes assayed by the quenching of the fluorophore calcein trapped inside the vesicles. Mn^{2+} indicates addition of manganese, Val. of valinomycin. (**c**) In liposomes devoid of protein no transport is observed. (**d**) Manganese transport into proteoliposomes in conditions where Na^+ was replaced by choline. (**e**) Mn^{2+}-dependent transport of H^+ assayed by the quenching of the pH-dependent fluorophore ACMA at an initially symmetric pH of 7.2. From left to right: Time dependent changes of the fluorescence signal of ACMA after addition of Mn^{2+} and valinomycin to liposomes devoid of protein. H^+ transport in proteoliposomes containing EcoDMT at an intravesicular buffer concentration of 6 mM and 60 mM, and in the presence of 2 mM intravesicular EDTA. (**b**–**e**) Traces are shown in unique colors with outside Mn^{2+} concentrations (μM) indicated.

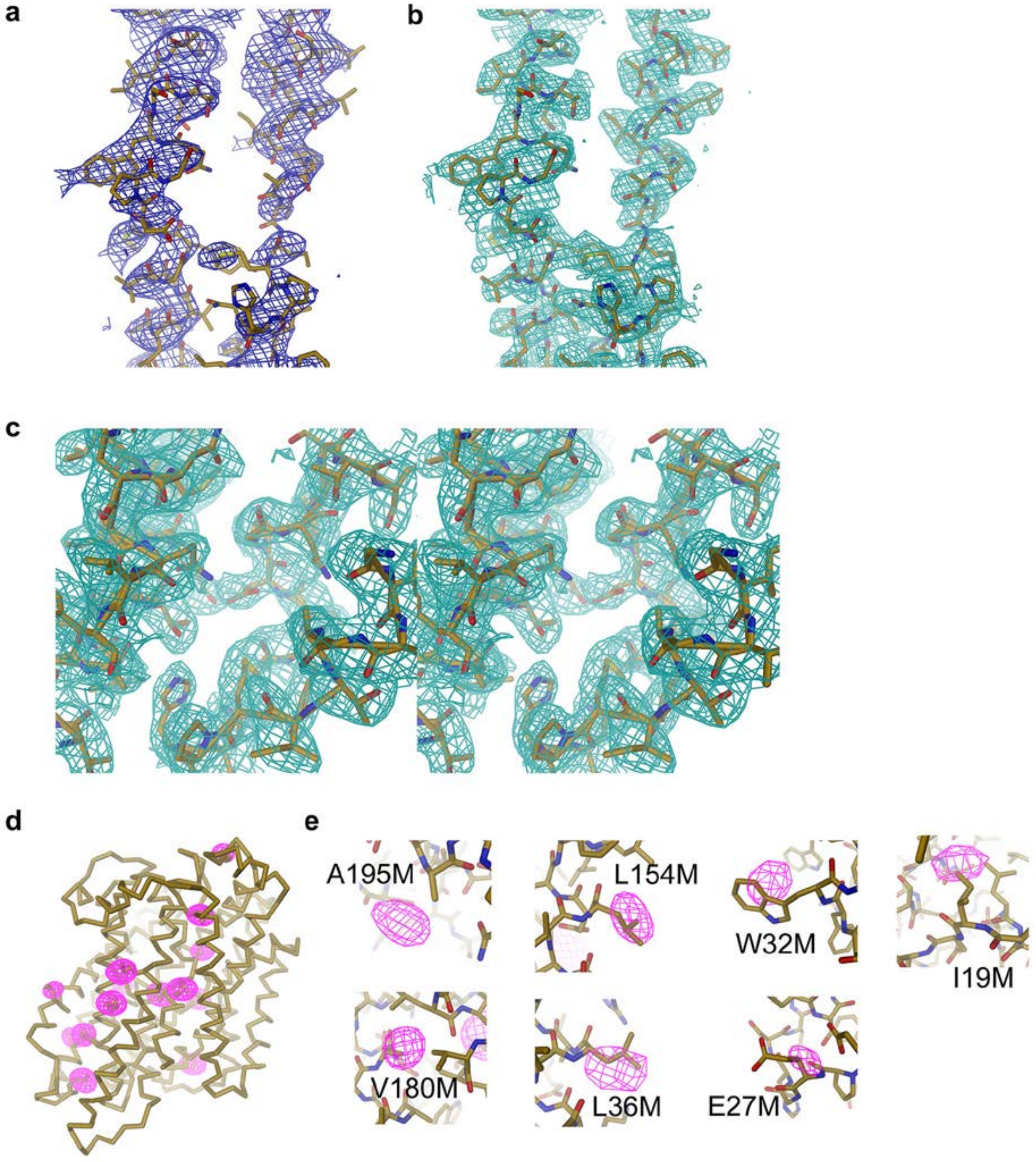

Supplementary Figure 3

Electron density.

(**a**) Experimental electron density (calculated at 3.6 Å with solvent flattened MAD phases and contoured at 1σ, blue mesh) is shown superimposed on α-helices 1 and 6 of the refined model. (**b**) $2F_o$–F_c electron density of the same region is shown superimposed on the refined model**.** (**c**) Stereo representation of the ion-binding site viewed from the extracellular side with $2F_o$–F_c electron density superimposed**.** (**b**,**c**)**,** Sharpened electron density (b=120) was calculated with model phases at 3.3 Å, contoured at 1σ and is shown as cyan mesh. (**d**) Anomalous difference electron density (calculated at 3.6 Å and contoured at 6σ, magenta mesh) from data of crystals containing Se-Met derivatized WT is shown superimposed on a Cα-trace of EcoDMT. Methionine side chains are displayed as sticks. (**e**) Anomalous difference electron density from data of crystals containing Se-Met derivatized point mutants in the vicinity of introduced methionine positions (calculated at 4.5 Å and contoured at 4.5σ, magenta mesh). The refined WT structure is shown as sticks.

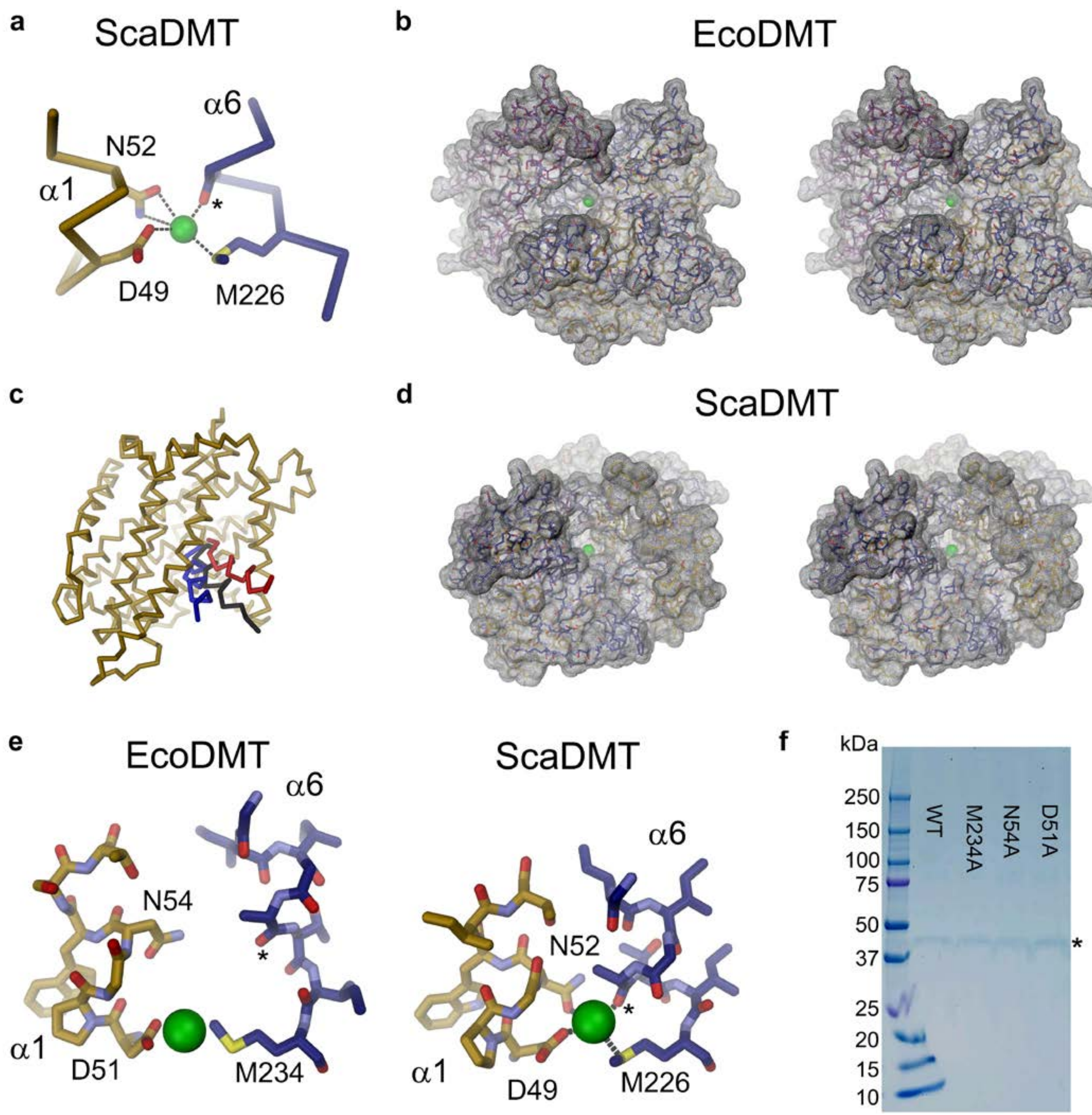

Supplementary Figure 4

Structural features of ScaDMT and EcoDMT and reconstitution efficiency of EcoDMT ion-binding site mutants.

(**a**) Transition-metal ion coordination within the ion-binding site of ScaDMT. The protein is shown as Cα trace, selected main chain and side chain atoms as sticks. Interactions with the Mn^{2+} ion (green sphere) are indicated by dashed lines. (**b**) Stereo representation of EcoDMT viewed from the extracellular side. The protein is shown as sticks with the molecular surface superimposed as grey mesh. A modeled Mn^{2+} ion is shown as green sphere. (**c**) Ribbon representation of ScaDMT with the modeled helix α1, which was not present in the crystallized construct, shown in black. The corresponding helices from the superimposed structures of LeuT (PDB ID 3TT3) and vSGLT (PDB ID 3DH4) are shown for comparison in red and blue respectively. (**d**) Stereo representation of ScaDMT (containing helix α1 in a modeled conformation) viewed from the intracellular side. The protein is shown as sticks, the molecular surface is superimposed as grey mesh. A crystallographically defined Mn^{2+} ion is shown as green sphere. (**e**) Substrate-binding sites of EcoDMT (left) and

ScaDMT (right). Proteins are shown as sticks. Mn^{2+} (green sphere) in the EcoDMT structure was modeled, in ScaDMT it is placed at the experimentally defined position. Binding-site residues in EcoDMT are labeled. In (**a**,**e**) * indicates a carbonyl group of the backbone of α-helix 6a that is involved in Mn^{2+} interactions in ScaDMT. (**f**) SDS-PAGE gel of EcoDMT (*) extracted with the detergent DM from equivalent amounts of proteoliposomes shows a comparable reconstitution efficiency for WT and the metal ion-binding site mutants D51A, N54A and M234A.

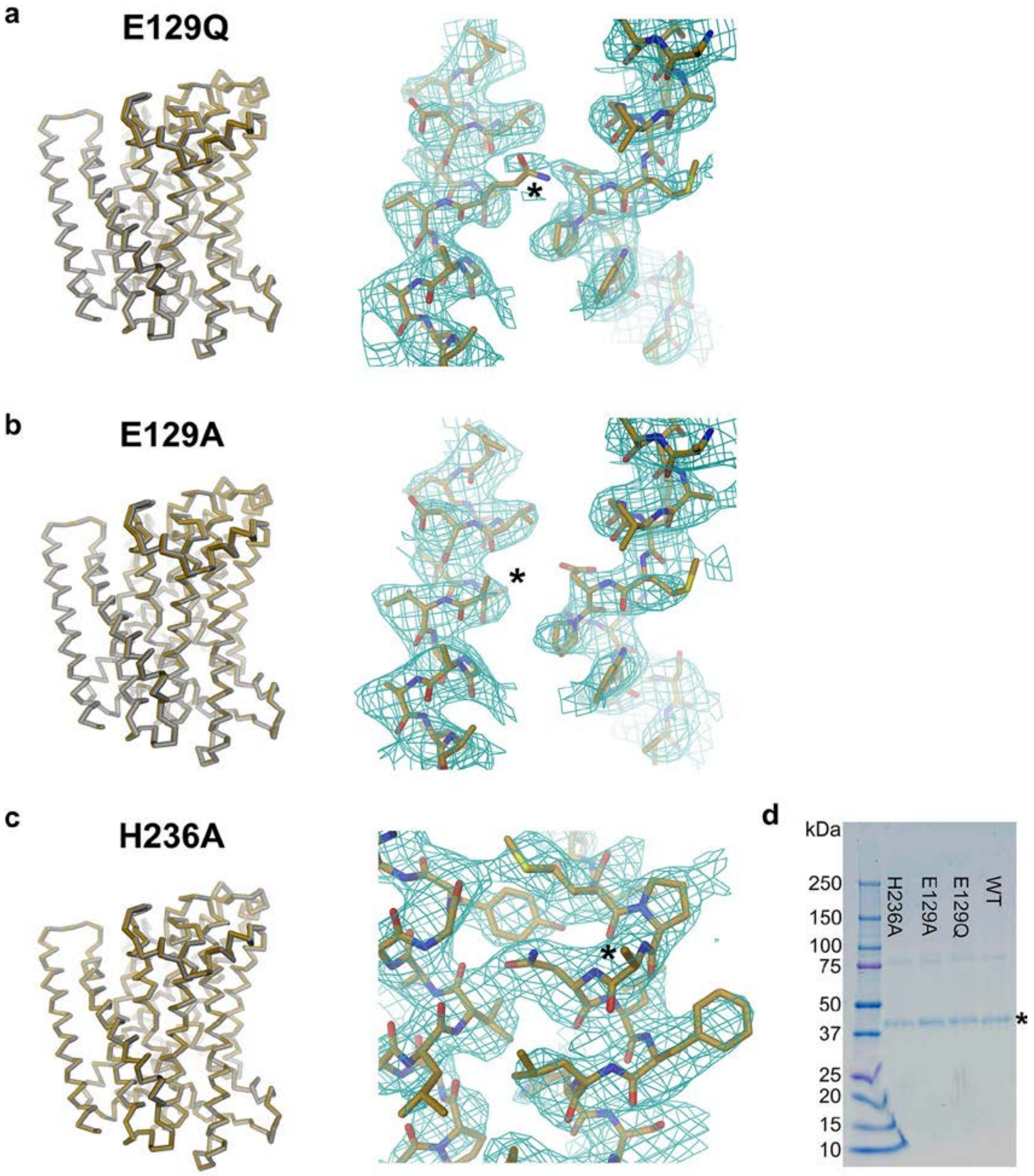

Supplementary Figure 5

Structure and reconstitution efficiency of potential proton acceptor mutants of EcoDMT.

(**a**) Left: Superposition of a Cα-trace of WT (grey) and the mutant E129Q (beige). Right: $2F_o$–F_c electron density calculated at 3.6 Å superimposed on the refined E129Q structure. (**b**) Left: Superposition of a Cα-trace of WT (grey) and the mutant E129A (beige). Right: $2F_o$–F_c electron density calculated at 3.9 Å superimposed on the refined E129A structure. (**c**) Left: Superposition of a Cα-trace of WT (grey) and the mutant H236A (beige). Right: $2F_o$–F_c electron density calculated at 3.7 Å superimposed on the refined H236A structure. (**a**–**c**) Sharpened $2F_o$–F_c Electron densities (b=120, contoured at 1σ and shown as cyan mesh) were calculated with phases from the refined models. In (a–c), an asterisk indicates the mutated side chain. (**d**) SDS-PAGE gel of EcoDMT (*) extracted with the detergent DM from equivalent amounts of proteoliposomes shows a similar reconstitution efficiency for WT and mutants of potential proton acceptors.

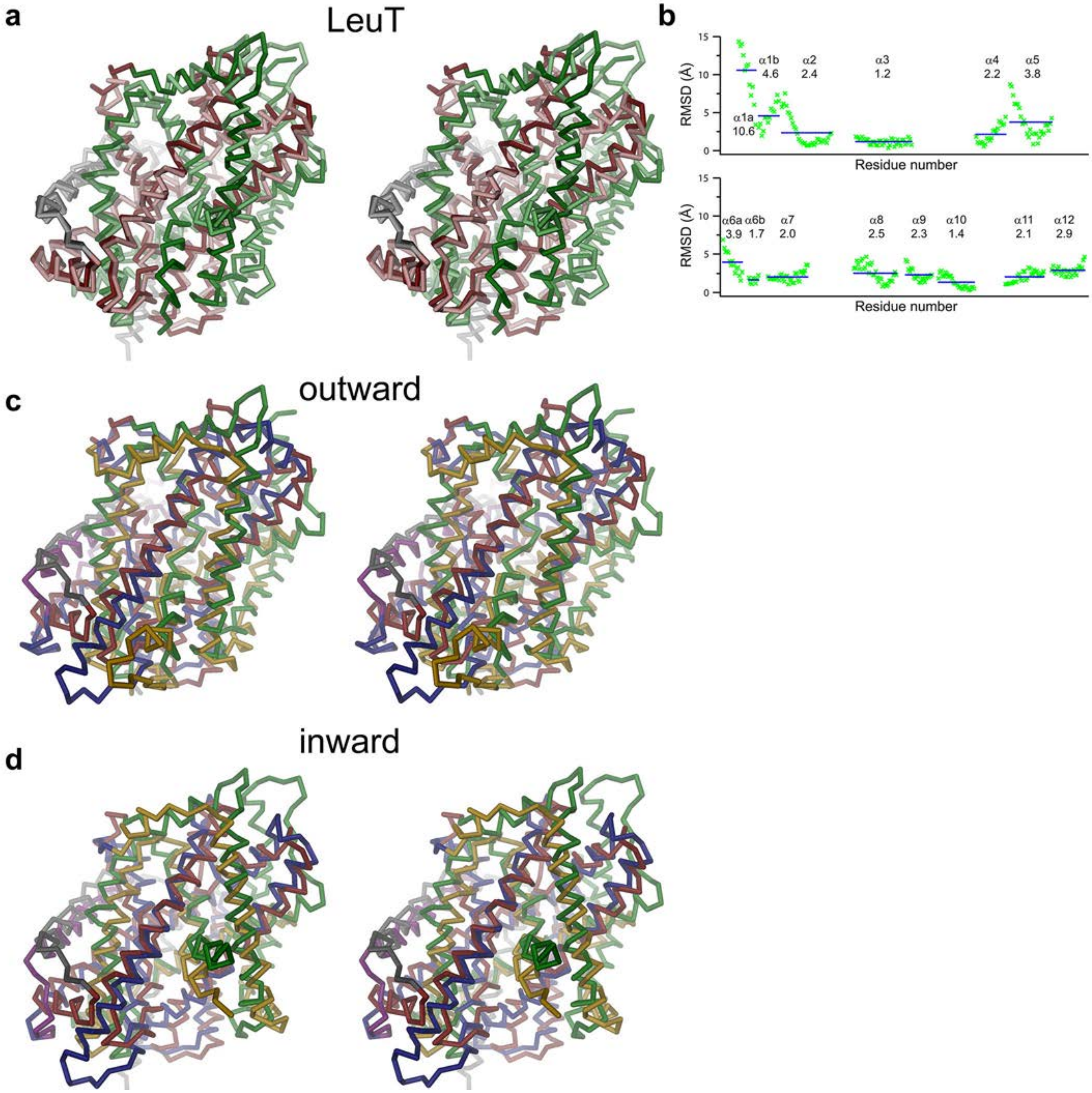

Supplementary Figure 6

SLC11-LeuT comparison 1.

(**a**) Stereo view of a superposition of inward-facing (PDB ID 3TT3) and outward-facing (PDB ID 5JAE) conformations of LeuT. The proteins are shown as Cα-traces. The view is from within the membrane with the extracellular side on top. In both structures, the N-terminal domain is colored in green, the C-terminal domain in red and α-helices 11 and 12 in grey but lighter colors are used for the inward-facing conformation. (**b**) RMSD of Cα positions calculated from a least-square superposition of equivalent regions of both conformations of LeuT. The residue number is plotted on the x-axis. Blue lines and numbers show averages for each transmembrane segment. Stereo views of superpositions of (**c**) EcoDMT and the outward-facing conformation of LeuT and (**d**) ScaDMT (PDB ID 5M94) and the inward-facing conformation of LeuT. The coloring is as in **a** and **Fig. 2a**.

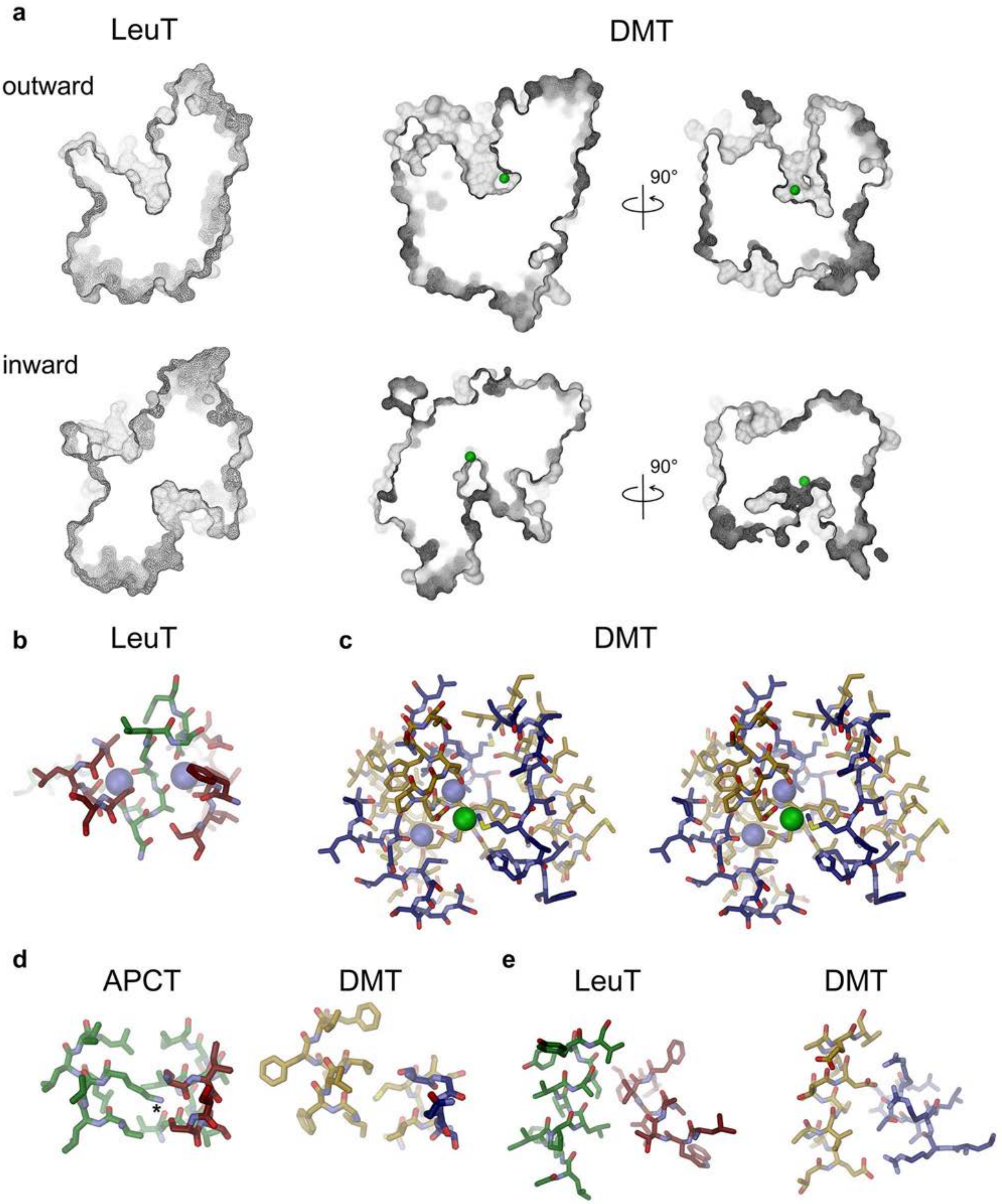

Supplementary Figure 7

SLC11-LeuT Comparison 2.

(**a**) Aqueous cavities in inward and outward conformations of amino acid and transition-metal ion transporters. The molecular surfaces are represented as grey mesh. Slices allow a view of the aqueous cavities leading to the substrate-binding sites that are indicated by

bound Mn^{2+}(green) for EcoDMT (top) and ScaDMT (bottom). The relationship between two different views of DMT structures is indicated. (**b**) Region around the two Na^+-binding sites in LeuT (PDB ID 3F3E). Sodium is represented as light-blue sphere. (**c**) Stereo view of the equivalent region of EcoDMT. The Na^+ positions (light blue spheres) were obtained from a superposition of the LeuT structure and are shown as reference. The green sphere represents a modeled Mn^{2+}. (**d**) Region around a lysine (*) of the proton-coupled amino acid transporter APCT (PDB ID 3GI8) that was implicated to play a role in H^+ transport (left). The equivalent region in EcoDMT does not contain a protonatable residue (right). (**e**) Sections of LeuT (left) and EcoDMT (right) around α-helices 3 and 9. In SLC11 transporters, this region contains conserved acidic and basic residues that constitute a potential H^+-pathway. In LeuT, the equivalent region is lined with hydrophobic residues. (**b**–**e**) The proteins are shown as sticks, the coloring is as in **Fig. 2a** and **Supplementary Fig. 6a**.

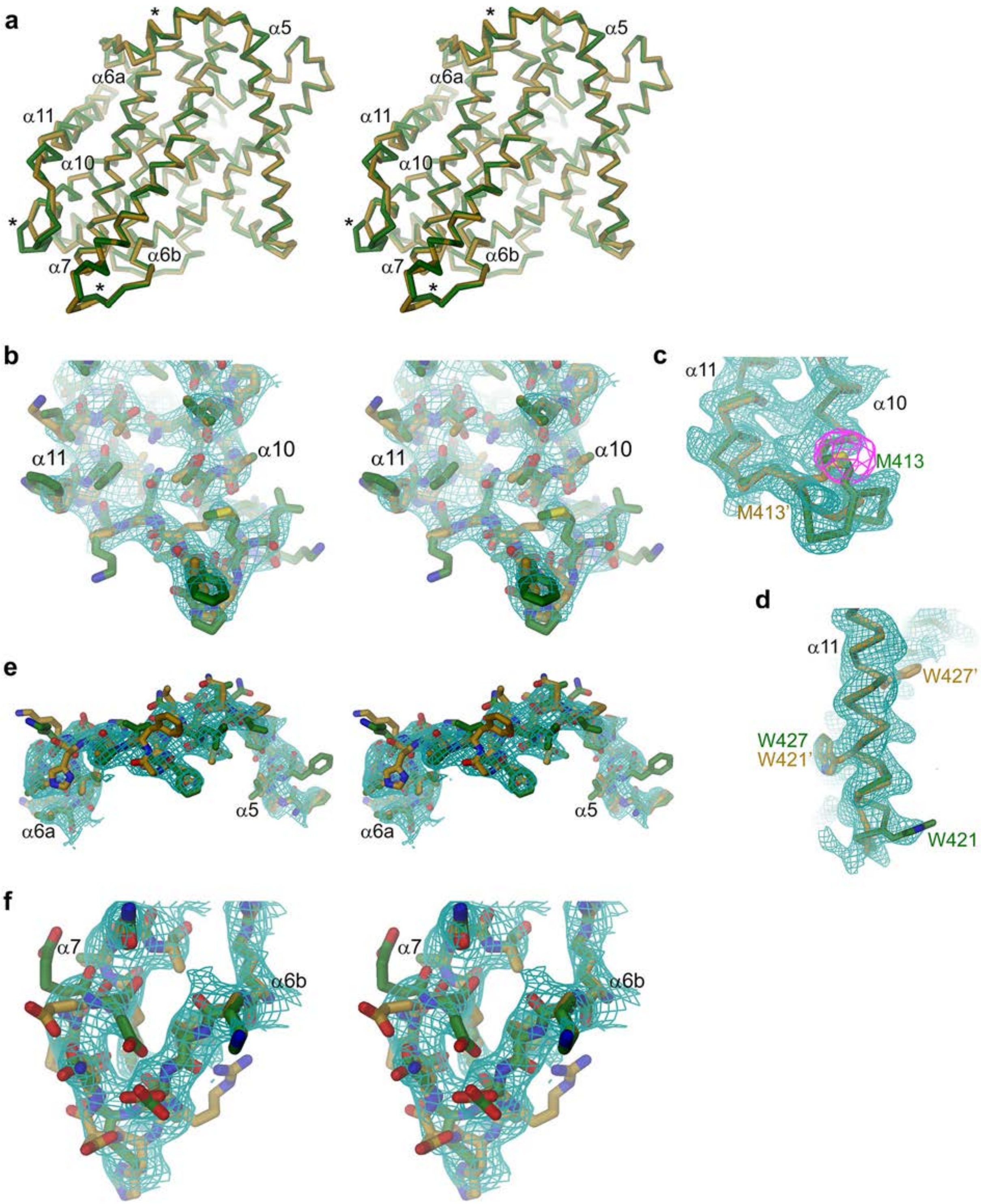

Supplementary Figure 8

Correction of the ScaDMT structure.

Structural similarities between EcoDMT and ScaDMT have allowed the detection of local errors in the original ScaDMT structure (PDB IDs 4WGV and 4WGW). The incorrect tracing of the loop connecting α-helices 10 and 11 has caused a shift in the register of the terminal α-helix 11 by 6 residues. The region is located at the periphery of the protein. Local errors in the loops connecting α-helices 5

and 6a and α-helices 6b and 7 did not cause a shift in the register of the neighboring transmembrane α-helices. In no case have these errors affected any conclusions drawn from the structure. The corrected coordinates were deposited with the PDB under accession codes (5M94 and 5M95). Carbon atoms of the original structure (PDB ID 4WGV) are shown in beige and of the corrected structure in green. (**a**) Stereo view of a superposition of Cα traces of the original and corrected ScaDMT structure. Selected helices are labeled, the regions containing the incorrectly traced loops are indicated by an asterisk. (**b**) Close up of the mistraced region between α-helices 10 and 11. (**c**) Cα trace of the loop connecting α-helices 10 and 11. Anomalous difference electron density (calculated at 3.5 Å and contoured at 7 σ) from data of crystals grown from Se-Met derivatized WT protein is shown as magenta mesh, the side chain of Met413 as sticks. (**d**) Close up of α-helix 11 with the side chain of two tryptophan residues shown as sticks. (**c**-**d**) Selected residues are labeled. Positions corresponding to the original structure are marked with an apostrophe (**e**) Close up of the mistraced region between α-helices 5 and 6a. (**f**) Close up of the mistraced region between α-helices 6b and 7. (**b**-**f**) $2F_o–F_c$ density (calculated at 3.1 Å and contoured at 1σ) is shown as cyan mesh superimposed on the models.

Supplementary Table 1 Primers

**EcoDMT construct**	**primers[a]**	**purpose**
WT	forward: ATATATGCTCTTCTAGTGAAACTCAATCCCAAACTATGACTAGA<br>reverse: TATATAGCTCTTCATGCTTTTTGCTTTGGATGGGTAAAACGACG	FX cloning
H236A	forward: CATTATGCCCGCCAATCTTTTCCTTCATTCCGCCATTTCC<br>reverse: GGCGGGCATAATGGTTGCCCCAATAATCCCTAAGCTGG	mutagenesis
E127Q	forward: TTGCCCAAGTTATCGGAGCCGCCATTGCCCTTTACC<br>reverse: ATAACTTGGGCAATATCTGTCGCCATGATGGCTAATTCTGTG	mutagenesis
E127A	forward: TTGCCGCAGTTATCGGAGCCGCCATTGCCCTTTACC<br>reverse: ATAACTGCGGCAATATCTGTCGCCATGATGGCTAATTCTGTG	mutagenesis
I19M	forward: CCTGAAGGACATTCCTTTTGGAAAACTCTCCTGGCTTATTCTGG<br>reverse: ATGTCCTTCAGGGACTTCTACGGTACTGTTCATGTCGCTC	mutagenesis
E27M	forward: CCTATGGGACATTCCTTTTGGAAAACTCTCCTGGCTTATTCTGG<br>reverse: CCTGAAGGACATTCCTTTATGAAAACTCTCCTGGCTTATTCTGG	mutagenesis
W32M	forward: CCTGAAGGACATTCCTTTATGAAAACTCTCCTGGCTTATTCTGG<br>reverse: ATGTCCTTCAGGGACTTCTACGGTACTGTTGATGTCGCTC	mutagenesis
L36M	forward: CCTGAAGGACATTCCTTTTGGAAAACTATGCTGGCTTATTCTGG<br>reverse: ATGTCCTTCAGGGACTTCTACGGTACTGTTGATGTCGCTC	mutagenesis
L154M	forward: TAATGGATGTCTTCTTACTCTTGCTTCTTAACCGAATCG<br>reverse: GAAGACATCCATTACCGTAATCACAACGGCTAGG	mutagenesis
V180M	forward: ATGATTCTCTTCGTCTTCCTTTATCAAATTATTCTCTCTCAGC<br>reverse: ACGAAGAGAATCATAAAAATTAGGCAGACGACTAGG	mutagenesis
A196M	forward: AATGTGGCATCAAGTAGCTAAGGGTCTCATCCCTTCC<br>reverse: CTTGATGCCACATTGGCTGAGAGAGAATAATTTGATAAAGG	mutagenesis

[a]primer sequences are depicted in 5’-3’ direction
